# Tracking metabolic responses based on macronutrient consumption: A comprehensive study to continuously monitor and quantify dual markers (cortisol and glucose) in human sweat using WATCH sensor

**DOI:** 10.1002/btm2.10241

**Published:** 2021-07-29

**Authors:** Madhavi Pali, Badrinath Jagannath, Kai‐Chun Lin, Devangsingh Sankhala, Sayali Upasham, Sriram Muthukumar, Shalini Prasad

**Affiliations:** ^1^ Department of Bioengineering University of Texas at Dallas Richardson Texas USA; ^2^ Department of Electrical Engineering The University of Texas at Dallas Richardson Texas USA; ^3^ EnLiSense LLC Allen Texas USA

**Keywords:** biosensors, cortisol, glucose, macronutrients, metabolism, passive sweat sensing technology

## Abstract

**W**earable **A**wareness **T**hrough **C**ontinuous **H**idrosis (WATCH) sensor is a sweat based monitoring platform that tracks cortisol and glucose for the purpose of understanding metabolic responses related to macronutrient consumption. In this research article, we have demonstrated the ability of tracking these two biomarkers in passive human sweat over a workday period (8 h) for 10 human subjects in conjunction with their macronutrient consumption. The validation of the WATCH sensor performance was carried out via standard reference methods such as Luminex and ELISA This is a first demonstration of a passive sweat sensing technology that can detect interrelated dual metabolites, cortisol, and glucose, on a single sensing platform. The significance of detecting the two biomarkers simultaneously is that capturing the body's metabolic and endocrinal responses to dietary triggers can lead to improved lifestyle management. For sweat cortisol, we achieved a detection limit of 1 ng/ml (range ∼1–12.5 ng/ml) with Pearson's “*r*” of 0.897 in reference studies and 0.868 in WATCH studies. Similarly, for sweat glucose, we achieved a detection limit of 1 mg/dl (range ∼ 1–11 mg/dl) with Pearson's “*r*” of 0.968 in reference studies and 0.947 in WATCH studies, respectively. The statistical robustness of the WATCH sensor was established through the Bland–Altman analysis, whereby the sweat cortisol and sweat glucose levels are comparable to the standard reference method. The probability distribution (*t*‐test), power analysis (power 0.82–0.87), *α* = 0.05. Mean absolute relative difference (MARD) outcome of ˷5.10–5.15% further confirmed the statistical robustness of the sweat sensing WATCH device output.

## 1 INTRODUCTION

According to the CDC National Center for Health Statistics, the prevalence of obesity in adults was 42.4% in 2018. With the hectic lifestyle combined with the decrease in the quality of food being consumed, these numbers are expected to skyrocket by the end of this decade.^1^ This prevalence has led to World Health Organization (WHO) declaring obesity as a major unmet public health problem.^2^, ^3^ Obesity is linked to several pathological disorders including hypertension, type 2 diabetes mellitus, cardiovascular diseases, cancer, respiratory system abnormalities, sleep disorders, and metabolic disorders.^4^ Specifically, this obesity pandemic has resulted in a dramatic increase in cases of type 2 diabetes mellitus and cardiovascular diseases. However, the outcomes of being obese do not often lead to complications resulting in the development of lifestyle disorders. This case‐by‐case variation in the disease progression is caused due to a complex interplay of genetic and environmental factors that contributed to obesity in the first place. The origins of this disease can be either due to genetic predisposition or due to dietary intake combined with a sedentary lifestyle.^5^ One of the factors that exacerbate the outcomes of obesity includes dietary fat and carbohydrate intake.^6^ Reports suggest that the main cause of obesity is the imbalance between energy (food) intake and energy expenditure, along with genetic and environmental contributed effects. There is significant evidence that indicates that cortisol might be a major component of the factors contributing to the development of obesity.^7^

Cortisol is a glucocorticoid and plays an important role in the context of macronutrients. It supports energy regulation by selecting the right type of macronutrient (carbohydrate, fat, or protein) that the human body needs to meet the physiological demands placed on it. Mainly, the effects of macronutrient content are considered to be mediated by alterations in cortisol action. Examples include studies that have reported a direct association between cortisol levels and calorie intake in population groups of women.^8^ Previous research has shown that glucose intake amplified the cortisol response to psychosocial stress, while low blood glucose (BG) levels prevented the stress‐induced activation of the hypothalamus–pituitary–adrenal (HPA) axis.^9^ Chronically high BG levels along with insulin suppression could lead to cells that are starved of glucose. Glucose modulation in conjunction with cortisol's effect on appetite can create a craving for high‐calorie foods, leading to overeating, which ultimately results in obesity in the long run. Several dietary and endogenous factors affect the maintenance of an appropriate level of glucose and cortisol in human blood. Cortisol acts on the glucose amount by activating glycogen stores in the liver, reducing the oxidation of glucose, stimulation of lipolysis, and significant enhancement or elevation of gluconeogenesis in response to severe amino acid imbalance. Mobilization of glucose reserves is particularly important in the case of stress inducing situations like endurance training due to prolonged effort.^10^ Several research studies have explored how macronutrient deficiency promotes type 2 diabetes in obese patients by triggering potential impairment of glucose metabolism, thereby causing insulin resistance^11^ A summary of this cycle is presented as Figure S1. This figure highlights the bidirectional relationship between chronic stress combined with increased glucose levels and metabolism. Increased obesity often puts the patient at a risk of developing type 2 diabetes, which then affects the HPA axis through inhibition of hippocampal receptors. The HPA axis stimulation leads to increase in cortisol levels, which stimulate gluconeogenesis, leading to increase in glucose level. In addition to this, it also contributes to metabolically induced insulin resistance that exacerbates the current diabetic condition.^12^ The dietary macronutrient content can alter the glucocorticoid metabolism as the result of increased cortisol release.^13^ The work done by Stimson et al. with obese participants who have varied dietary intake patterns has proven that the macronutrient contents are certainly mediated by alterations in cortisol release rates.^10^ This interelatibility of the molecules can be exploited to understand how dietary triggers and stress lead to the development of lifestyle disorders.

The global pandemic caused by COVID‐19 has led to living conditions that promote a sedentary lifestyle, and a low quality dietary intake; increasing our vulnerability to the COVID‐19 virus.^14^ The risk of cardiovascular diseases due to the SARS‐CoV‐2 pathogenic virus is believed to be aggravated by stress (affecting cortisol release rates) and sugar‐rich diets (affecting glucose release rate).^15^, ^16^ Furthermore, patients suffering from Diabetes Mellitus are at a higher risk for complications and death from COVID‐19. Thus, it highlights the need for self‐monitoring systems that will help with understanding the body's response to diet and keep a control on biomarker levels for this high‐risk population. Hence, there is an immediate need for technological interventions that can support on‐demand temporal monitoring of glucose and cortisol concerning the macronutrient uptake. Monitoring glucose and cortisol simultaneously and combinatorially provides meaningful insight into the physiological reactions (metabolic and endocrinal) to dietary intake and triggers related to the same. This is highly beneficial for improving lifestyle by understanding how macronutrient specifically relate to the body's functioning.

Current point‐of‐care diagnostics enable the measurement of glucose and cortisol separately at static time points. Glucose is typically measured through continuous glucose monitors and cortisol measurements are obtained from either salivary cortisol, urinary cortisol, or blood draws at specific time points.^17^, ^18^, ^19^ These measures are heterogeneous, in the sense, that they are obtained from different sensing platforms and are unable to provide a dynamic relationship between the two molecules as a function of macronutrient consumption. With the recent advancement in the development of point‐of‐need wearables, it has now become feasible to monitor both glucose and cortisol independently in a noninvasive manner. Human eccrine sweat has emerged to be the bio‐fluid of choice toward enabling the dynamic and on‐demand tracking of these biomarkers.^20^ However, the challenge is to perform sweat stimulation to such an extent that it collects sufficient volumes for performing real‐time monitoring of these target biomarkers. This volume insufficiency is a driving limitation of the process of collecting and analyzing the sample for biomarker quantification. Hence, in certain scenarios, noninvasive tracking lacks the accuracy needed for a real‐time and on‐demand measurements.^21^, ^22^, ^23^

Our group has pioneered in the ability to perform on‐demand measurements for several biomolecules present in passively expressed human eccrine sweat. Some of the recently published work includes detection of cortisol and DHEA for circadian diagnostics with a limit of detection of 0.1 ng/ml,^24^ monitoring IL‐1β, and CRP levels via SWEATSENSER to detect IBD flares,^25^ detecting glucose, alcohol, and lactate for lifestyle monitoring.^26^ These published works highlight the ability to track diagnostically relevant biomarkers in real‐time and in a noninvasive manner. Another example of the technological breakthrough presented by our group focuses on using low volumes of passive sweat for performing detection.^26^, ^27^, ^28^, ^29^, ^30^, ^31^This proves that there is a possibility in designing sensing platforms, which are capable of working with ultra‐low volumes of biofluid to perform biomarker detection. This makes it feasible to monitor large cohorts of the population who might have trouble generating a sweat sample passively. An example of this type of population includes people who may not be physically active because of compromised mobility due to injury, illness, or age. They cannot naturally produce significant sweat volumes that are typically needed to support sampling via micro fluidic sweat collectors. Their only recourse is the use of iontophoretic sweat stimulation systems, which have been shown to increase surface inflammation and are associated with many adverse epidermal effects. Hence, passive sweat‐based measurements circumvent all the previously mentioned challenges, enabling a hitherto unavailable opportunity to assess metabolite levels in an individualized manner, especially in users with a sedentary lifestyle. Additionally, with these advancements, there is a significant opportunity to expand the scope of testing into pediatric as well as geriatric populations. Passive sweat based sampling also ensures that the biomarker levels captured correspond to the actual concentrations as there is no suppression of the biomarker that is often associated with pilocarpine or other ionotophoretic sweat‐stimulation related activities.^32^ In this work, we have coupled the temporal, on‐demand measurements of glucose and cortisol as expressed in passive eccrine sweat in healthy but sedentary individuals and connected it to the individual's macronutrient consumption. We demonstrate an enabling technology that can equip users with the ability to identify the relationship between macronutrient consumption and the body's physiological response. We present WATCH studies with 10 human subjects to demonstrate the proof‐of‐technological feasibility of the passive sweat wearable platform. This works toward providing insights into the body's physiological response to dietary triggers. The relationship of cortisol and glucose with metabolism of macronutrients ensures that a single dual‐marker assay can provide a comprehensive evaluation of the user's health.

## 2 RESULTS

### 2.1 Evaluation of WATCH device performance

**FIGURE 1:**
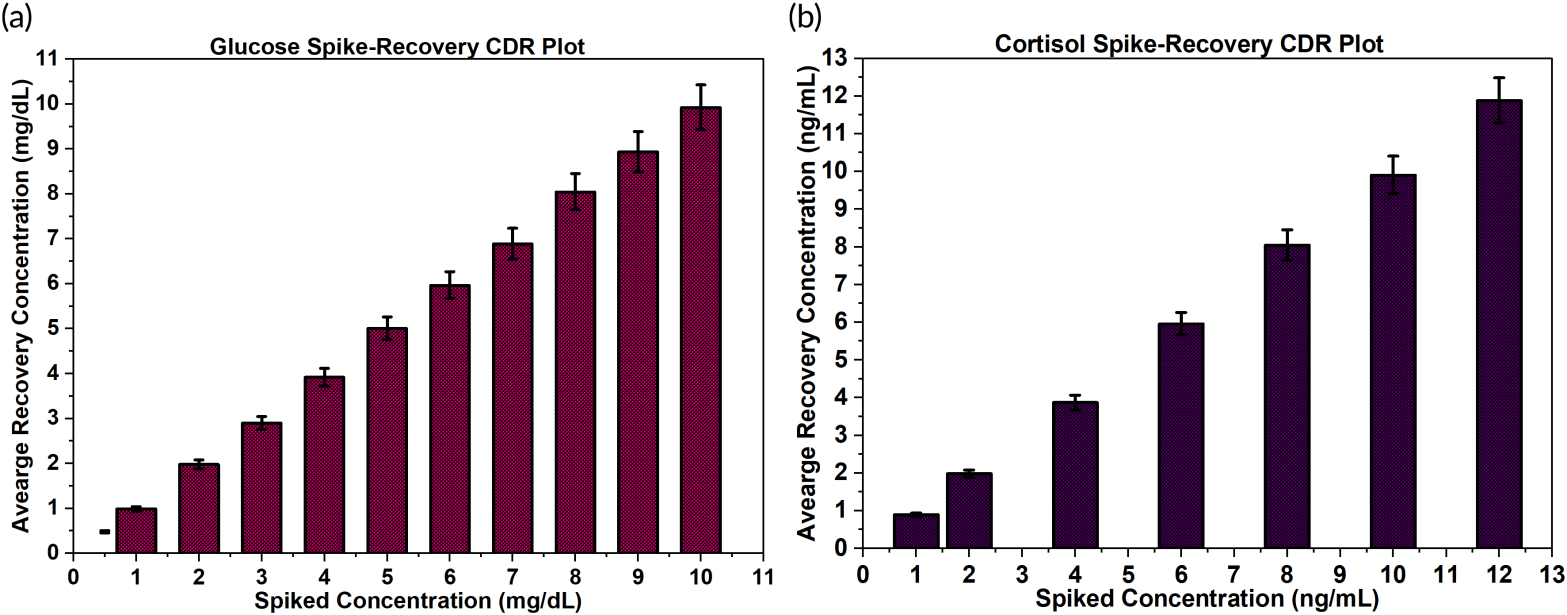
(a,b). Sensor surface dose–response demonstration for the detection and quantification of sweat glucose (a) and sweat cortisol. (b) Electrochemical measurements

Our device is an enabling prognosis technology to monitor affinity‐based interactions between target biomarkers and respective antibody probes on functionalized sensor surfaces.^33^ A dose–response calibration curve was developed to detect sweat glucose and cortisol by employing an in‐vitro benchtop SWEATSENSER device. To meet the required target concentration ranges that we are looking for in healthy human sweat, a series of dilutions were prepared for glucose (0–11 mg/dl) and cortisol analytes (0–12 ng/ml) in the sweat medium. A 4‐parameter fit was applied to build a calibration curve in response to obtained impedance results for both the biomarkers. The performance of the WATCH sensor was evaluated by conducting spike and recovery experiments for sweat glucose and cortisol. A series of log dilutions were spiked on functionalized sensor surfaces and the impedance response was recorded. The corresponding recovered concentration was computed from the developed calibration equation using the obtained impedance response. Figures 1a,b provide the spike‐recovery dose responses with standard deviation (SD) values of <0.05 for glucose and cortisol. This method of impedance‐based sensing was implemented to quantify both sweat biomarkers.

**FIGURE 2:**
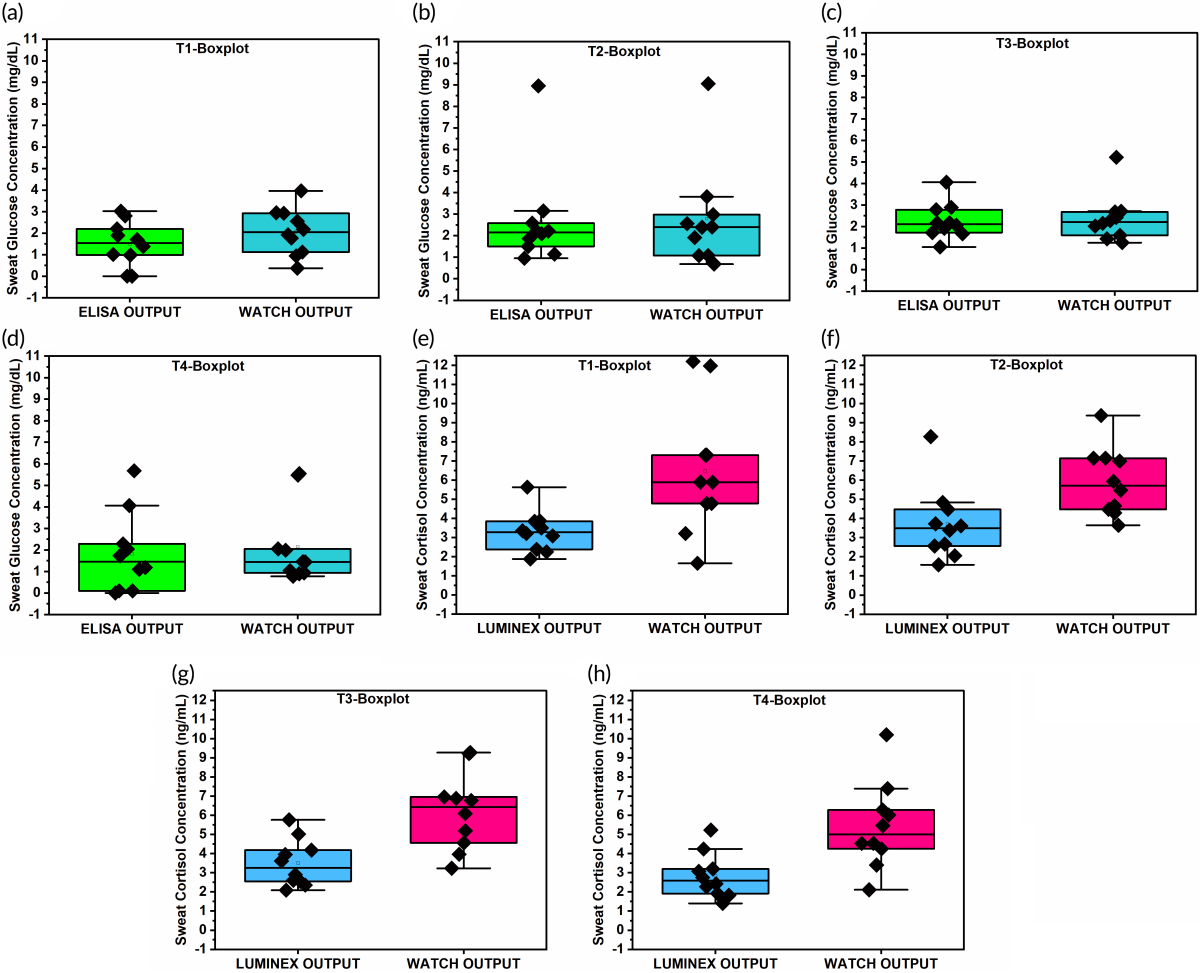
(a–d) Sweat glucose data Box plot representation at T1‐before breakfast (˷8:30 a.m.), T2‐after breakfast (˷9:30 a.m.), T3‐after lunch (˷1:30 p.m.), T4‐late evening (˷5:30 p.m.), discrete timepoint correlation of Reference standard versus WATCH output. (e–h): Sweat Cortisol data Box plot representation at T1‐before breakfast (˷8:30 a.m.), T2‐after breakfast (˷9:30 a.m.), T3‐after lunch (˷1:30 p.m.), T4‐late evening (∼ 5:30 p.m.), discrete timepoint correlation of reference standard versus WATCH output

The Box plot represented in Figure 2a–d for sweat glucose analysis includes standard reference measurements (ELISA) and WATCH measurements. These two analytical methods were employed on the same subject's sweat sample to understand the correlation of the detected concentrations to the testing environment. At discrete time‐points, two methods are linearly correlated with one another. The before breakfast (T1) sweat sample results for all 10 human subjects lie in the range of 0.09–3.31 mg/dl for ELISA,0.60–4.20 mg/dl for WATCH, and the rate of deviation between two methods is <0.93 mg/dl, that is, ˷0.12 mmol/L. The after breakfast (T2) sweat sample results for all 10 human subjects lie in the range of 1.01–9.10 mg/dl for ELISA, 0.85–9.35 mg/dl for WATCH, and the rate of deviation between 2 methods is <0.85 mg/dl, that is, ˷0.09 mmol/L. The postlunch (T3) glucose levels were in the range of 0.95–4.20 mg/dl for ELISA, 1.05–5.30 mg/dl for WATCH, and the rate of deviation is <0.7 mg/dl, that is, ˷0.08 mmol/L. The late evening (T4) levels for all 10 subjects lie in the range of 0.80–5.85 mg/dl for ELISA, 1.01–5.75 mg/dl for WATCH, and the rate of deviation between two methods is <0.85 mg/dl, that is, ˷0.09 mmol/L. The Box plot representation for sweat cortisol levels is illustrated in Figure. 2e–h, this includes the standard reference measurements (LUMINEX) and WATCH measurements. The sweat cortisol analysis is performed in a similar manner to the previously described sweat glucose testing and validation. The prebreakfast (T1) sweat sample results range from 1.88–5.63 ng/ml for LUMINEX, 1.65–12.45 ng/ml for WATCH, and the rate of deviation between 2 methods is <0.62 ng/ml, that is, ∼ 2.1 nmol/L. The after breakfast, (T2) sweat sample results lie in the range of 1.58–8.27 ng/ml for LUMINEX, 0.3.23–9.25 ng/ml for WATCH, and the rate of deviation between two methods is <0.75 ng/ml, that is, ˷2.50 nmol/L. The post lunch (T3) sweat sample results lie in the range of 2.09–5.76 ng/ml for LUMINEX, 3.15–9.15 ng/ml for WATCH, and the rate of deviation between two methods is <0.73 ng/ml, that is, ˷2.51 nmol/L. The late evening (T4) sweat sample results lie in the range of 1.40–5.23 ng/ml for LUMINEX, 2.11–10.35 ng/ml for WATCH, and the rate of deviation between 2 methods is <0.65 ng/ml, that is, ˷2.20 nmol/L. A summary of these values has been provided in Table S1.

**FIGURE 3:**
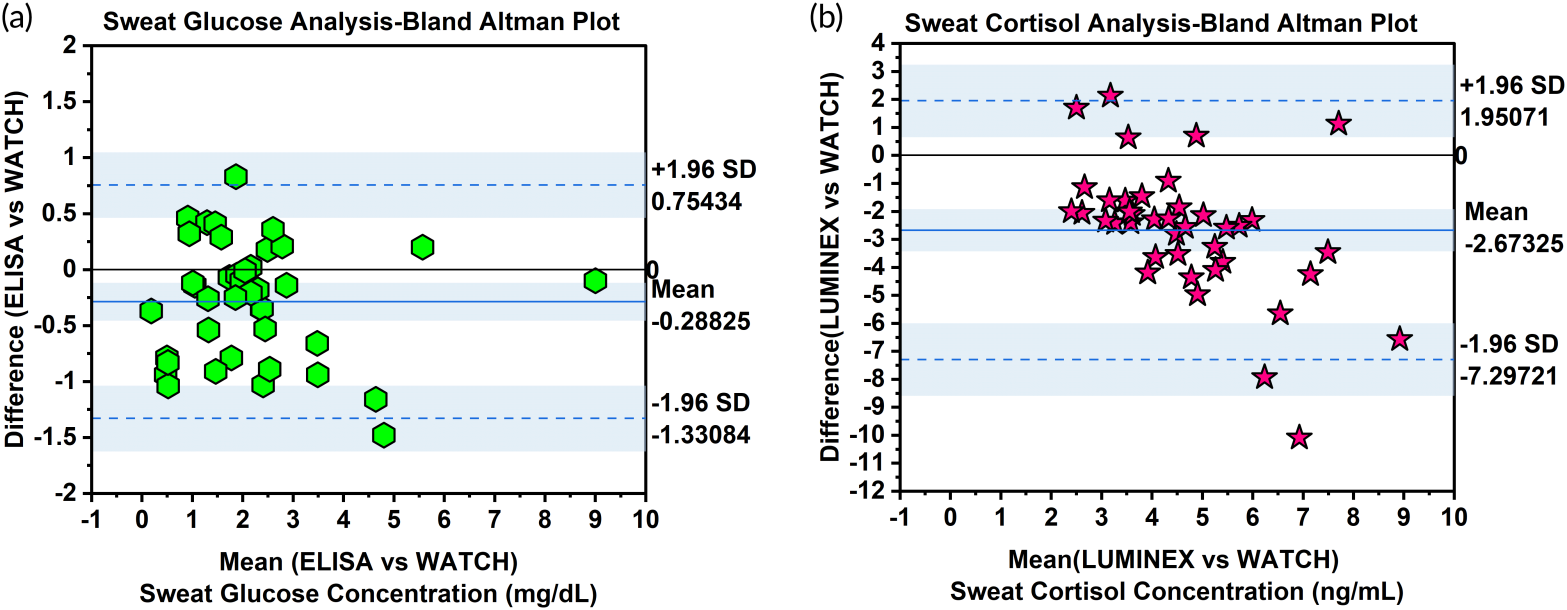
(a,b): A Bland Altman comparison plots on sweat glucose data: ELISA standard reference method versus WATCH Model prediction (10 data points each at 4 discrete time points—8.36 h study)

Bland–Altman analysis provides the mean bias or offsets of the value for all measured data points between the two analytical methods for the same variable. The *x*‐axis is an average, and the *y*‐axis is the difference between the reference method and the corresponding analytical method. The BA comparison plots were produced to interpret the level of agreement between reference versus WATCH method for each analyte. Figure 3a represent the BA comparison plots for sweat glucose concentration between ELISA versus WATCH data. The mean bias value is −0.28825 mg/dl, approximately close to 0 on the scale, indicating a minute difference between the two measurement methods. There are 2 or 3 data points in sweat glucose measurements that lie beyond ±1.96 SD. Sweat glucose data points are almost equally distributed between any pair of the methods presented with 1 or 2 outliers. The mean bias value of Reference versus WATCH indicates a close agreement between the two methods. Sweat cortisol data points using a reference method versus WATCH method was distributed with a lower mean bias of −2.67325 from a baseline of 0. This is indicative of the fact that reference methods are influenced by the selection of monoclonal antibodies and conjugated secondary biotinylated antibodies. The mean bias to a baseline of 0 indicating close agreement between the two analytical methods. Figure 3b represent the Bland–Altman comparison plots for sweat cortisol concentration between LUMINEX versus WATCH results. The mean bias value is representing below approximation to 0 scale. The majority of the datapoints lie under the ±1.96 SD range with the exception of 2 or 3 outlying data points. We observed a slightly negative mean bias in our WATCH measurements due to the amplified magnitude of sensitivity in reference methods like ELISA Optical Density output and LUMINEX magnetic bead‐based technology output. Several factors like detection modality, instrumentation, personnel hand skills, environmental conditions, and buffer medium influence the output. However, our output of concentration range lies within the detection limits and showed a corresponding linearity in change with the reference data. Hence, we were able to draw a relevant data‐driven conclusion.

### 2.2 WATCH performance: cross sectional observational study

This work presents the average values of all 10 healthy human cohort at 4 different time intervals to comprehend the trend of glucose and cortisol release rates in human sweat. The obtained concentration averages in case of sweat glucose are T1: 1.50 mg/dl, T2: 2.55 mg/dl, T3: 2.25 mg/dl, and falls to T4: 1.80 mg/dl for the ELISA reference method studies (Green). Similarly, the trend levels are at T1: 2.05 mg/dl, T2: 2.60 mg/dl, T3: 2.35 mg/dl, and T4: 2.10 mg/dl for WATCH results (BlueThe obtained concentration averages are T1: 3.35 ng/ml, T2: 3.55 ng/ml, T3: 3.5 ng/ml, and T4: 2.85 ng/ml for LUMINEX reference studies [purple].). Finally, the WATCH (red) levels are T1: 5.95 ng/ml, T2: 6.55 ng/ml, T3: 6.10 ng/ml, and T4: 5.45 ng/ml.

### 2.3 MARD analysis: WATCH performance in real‐world, use‐case environment

The Mean Absolute Relative Difference (MARD) analysis is a widely incorporated statistical measurement to evaluate accuracy of blood glucometer output in clinical trials.^34^ In this project, we applied the MARD analysis to monitor sweat glucose in healthy cohort as a pilot study. MARD is the average values of absolute error between WATCH device output versus Standard reference output. A minute or less change in percentages (<5–12%) indicates that the WATCH readings are in close agreement with Reference readings. If the change is large, then MARD percentage indicates greater discrepancies between the WATCH and reference measurements. Any differences in comparing two analytical methods can be accurately and precisely addressed by calculating MARD values.^35^ This statistical approach is widely incorporated to check the efficacy of medical devices performance in monitoring glucose levels. Hence, we applied MARD analysis in our research work to compete with the health market standards in sweat sensor making.

**FIGURE 4:**
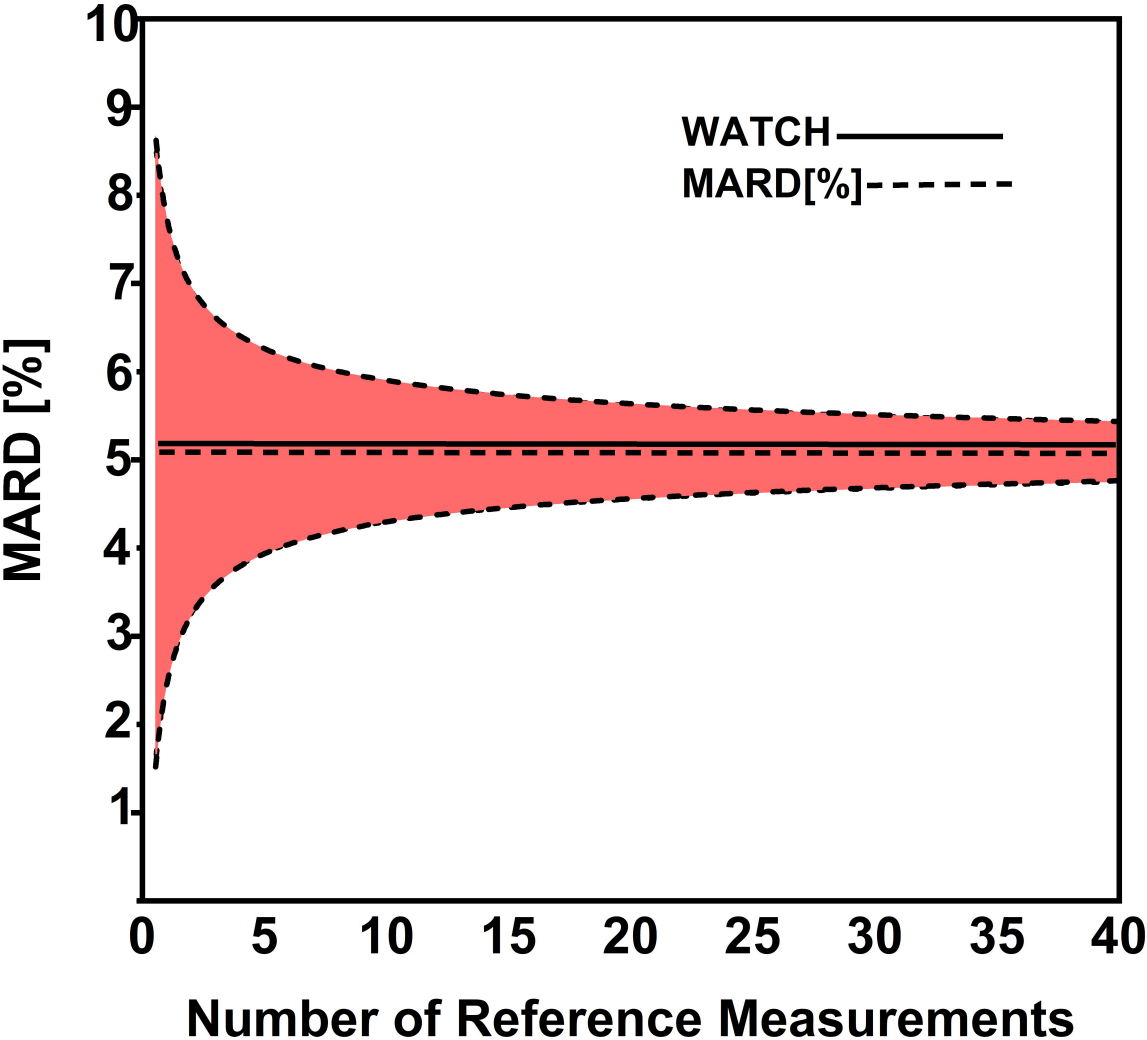
The number of paired points on the uncertainty of MARD analysis for the demonstrated WATCH sensor upper and lower bounds of the confidence interval (CI) with probability value *ϒ* = 0.95

The sweat glucose values obtained using the reference method can be deemed accurate within the acceptable error. A similar reasonable accuracy was also computed for the WATCH sensing technology. The continuous measuring output has desirable properties such as agreement with reference results and performance of the technology presented in this work was found to be consistent across subjects. The WATCH output lag deviation was found constant across the rise and fall of the sweat glucose values and this helped with building a static frame of reference for assessing accuracy using MARD. The expression for MARD is as given below in equations. Figure 4 shows good agreement between the reference and the WATCH measurements, exhibiting a low MARD % deviation for multiple points.

(1) $$\%\text{MARD}=100\%\times \frac{1}{N}\;\sum \frac{G_{\text{model}}-G_{\text{reference}}}{G_{\text{reference}}}\;$$

(2) $$\text{MARD}=\sum \frac{\left(\left|\mathrm{xi}-\bar{x}\right|\right)}{N}$$

In our study, the total time was around 8.5 h of continuous WATCH measurements and the reference measurements were collected at four discrete time points for each subject within this total time period. As shown in Figure 4, the first few points show higher MARD values, however with time, the MARD value decreases. This demonstrates that an increase in the number of comparison points results in good convergence between the measurement methods. Since MARD is a stochastic quantity, the area under the curve portion along *x*‐axis tends toward in convergence,^36^ and the continuous horizontal lines in Figure 4 show that the boundaries of uncertainty become tighter with an increasing number of paired points. For any given number of data points, published research has shown that normal expected MARD lies between 1 and ˷30%,^37^ with a confidence interval ranging between 0.95–0.97. In our study, the percent MARD value is 5.15% (Reference vs. WATCH sensor) with confidence interval (CI) value of 0.95.

### 2.4 Sweat biomarker trends: glucose and cortisol profile mapping using WATCH

**FIGURE 5:**
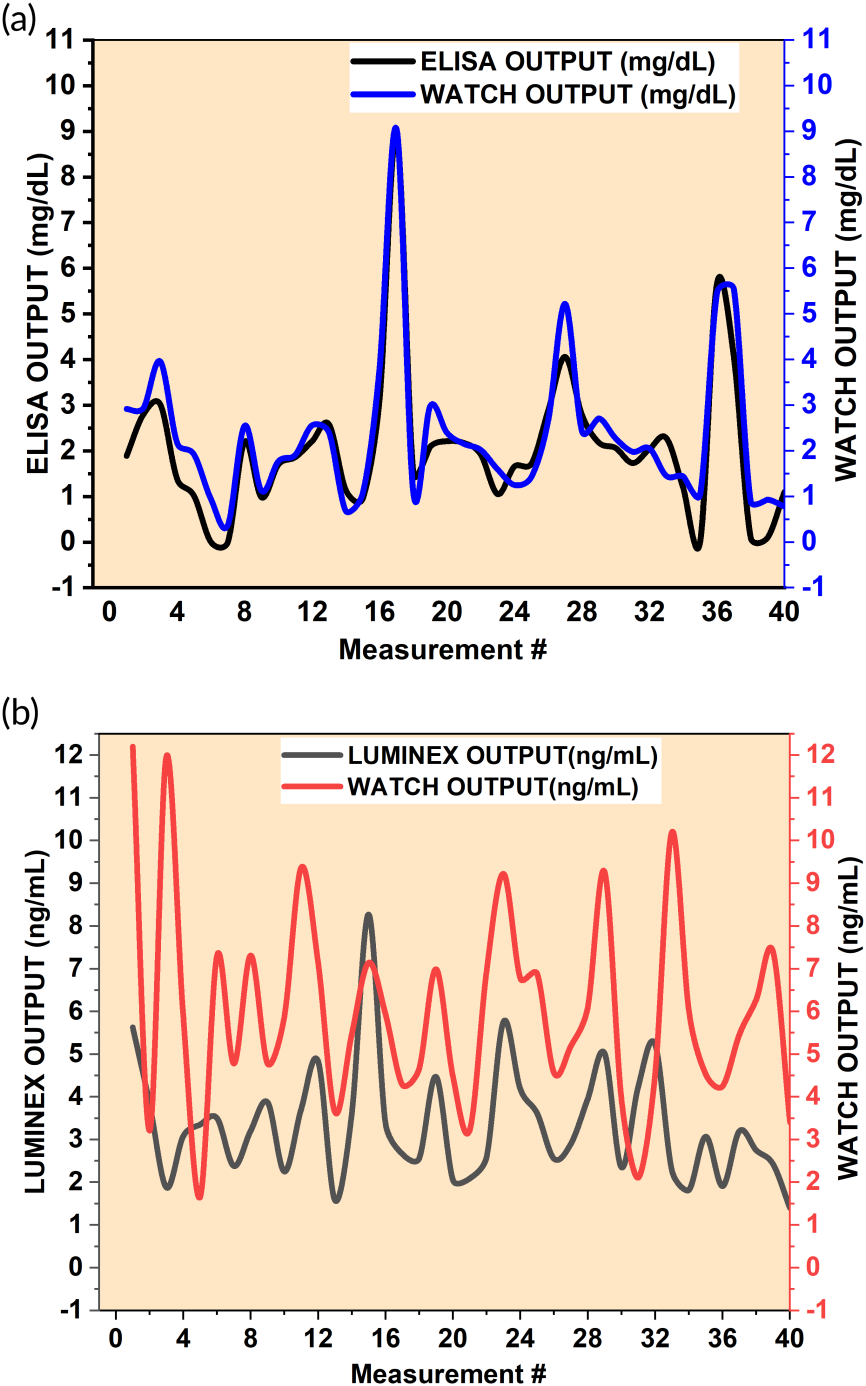
(a) The Sweat glucose trend profile (concentration range 0–11 mg/dl) for 10 human subjects, corresponding overlay curves represents ELISA reference method (black line) versus WATCH model prediction (blue line). (b) The sweat cortisol trend profile (concentration range 0–13 ng/ml) for all 10 human subjects at 4 time points and the corresponding overlay curves represents LUMINEX reference method (black line) versus WATCH model prediction (red line)

**FIGURE 6:**
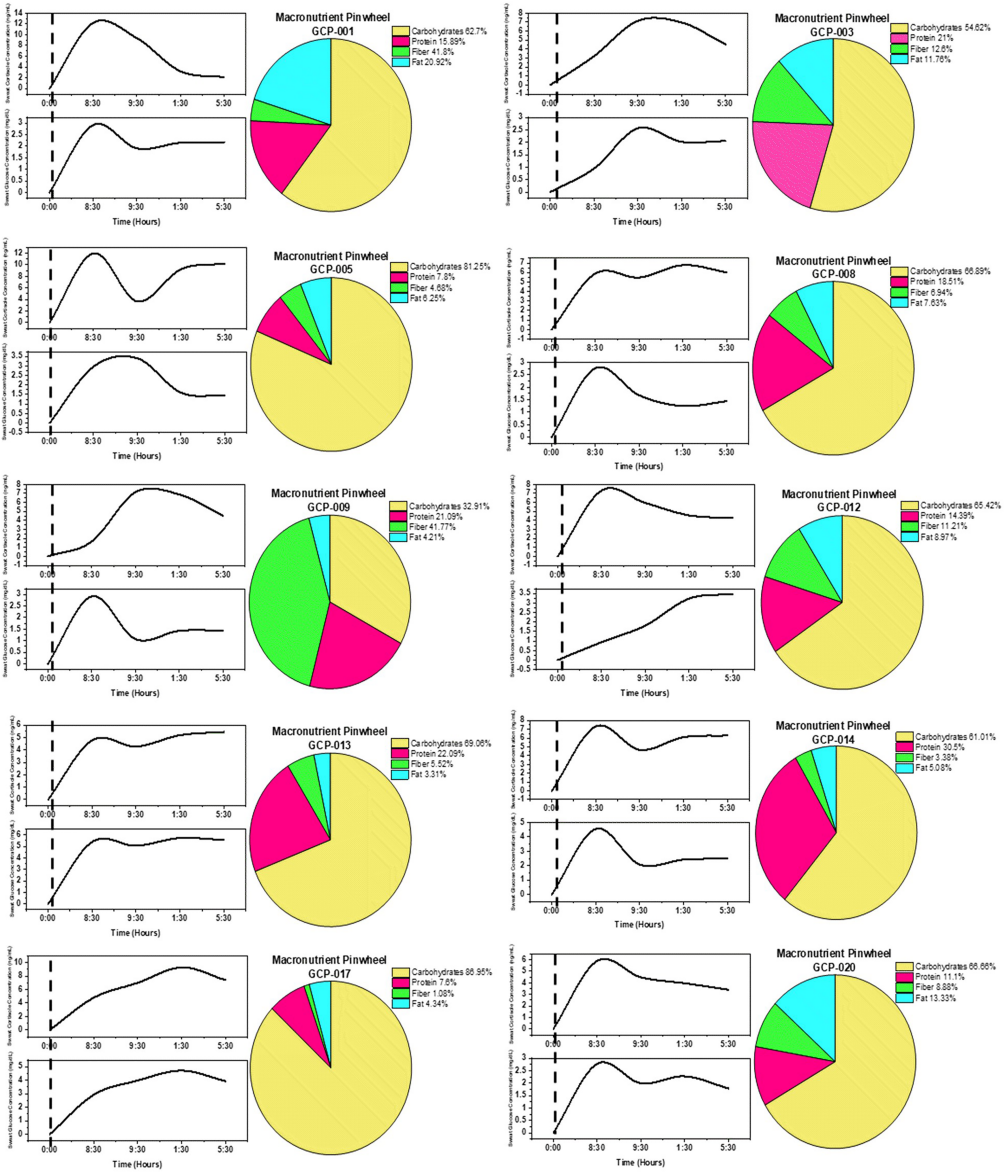
Macronutrient pinwheel pie chart description of participated human subjects (n = 10) in a time of 8‐h 35 min, continuous monitoring and their food consumption details such as the percentage of carbohydrates, protein, fat, and fiber contents shown next to the curve graphs

Discrete timepoint based sweat glucose profiles for all 10 human subject cohort were matched by comparing reference analytical method (ELISA) based results to the WATCH sensor results. Similarly, sweat cortisol profiles for all 10 human subject cohort were matched by comparing reference analytical method (LUMINEX) results to the sensor results. Figure 5a represents the measured glucose concentrations in sweat. Figure 5b represents the measured cortisol concentrations in sweat samples obtained from the volunteers. Figure 6 provides the information of food intake with amounts of carbohydrates, protein, fat, and fiber percentages in 10 subjects. The stick bar in black indicates the point where the food intake was recorded (approximately around 8:30 a.m.). The two curves representing glucose and cortisol levels in sweat were recorded over an 8‐h workday with the WATCH device. The trend lines upper (cortisol) and lower (glucose) in Figure 6 show us how both these metabolic markers fluctuate in the sweat throughout the day. The macronutrient pinwheel information for each subject is demonstrated next to the glucose and cortisol plots.

### 2.5 Probability distribution and power analysis

Independent sample *t*‐test was performed between reference and the sensor data to validate the distribution probability and to compare mean distribution between the analytical methods. The total mean measurements at each time point are accompanied with a sample size of 40 (10 subjects × 4‐time points) and their respective variability in concentrations are presented in the supplementary section. These values confirm that the differences in the distributions of the measurements by method are statistically insignificant. Regarding reporting the biomarker levels among the three methods of data collection for the two sweat markers, it can be observed that the power lies between 0.82 and 0.87 with an α of 0.05. Based on this study, we are confident that there is a possibility of achieving a power value of >0.93 in the future work by increasing the cohort of participants in the study. This would help healthcare professionals in understanding the relationship between macronutrient consumption versus fluctuations in glycemic‐adrenal indices by relying on measurements from the WATCH sensing platform.

## 3 DISCUSSION

The work focuses on observing and understanding the impact of major macronutrient groups (carbohydrates, proteins, fats etc.) on glucose and cortisol expression in healthy individuals with our sensing technology (WATCH device). Both glucose and cortisol are involved in pathways that focus on maintaining homeostasis that is linked to energy intake via food consumption. Detection of physiologically relevant range of glucose (hypo‐ to hyperglycemic range: 0.01–200 mg/dl) in perspired human sweat was successfully demonstrated by Bhide et al.^26^ This work is a first demonstration in the sweat sensing realm with a novel idea of leveraging impedimetric sensing to detect dual markers on a single sensor surface. Our attempt to incorporate the concept of macronutrient dietary intake monitoring with target marker detection further enhances the sensing modality by providing insight into the fluctuations that have the ability to alter the glucose‐cortisol response phenomenon. Our research group has previously demonstrated the Cortiwatch sensor that reports the physiological range of cortisol present in ambient sweat for circadian diagnostics.^38^ Based on this previous knowledge, we have further advanced the process of detecting cortisol levels in healthy human sweat using an ‐SH linked 5' Cortisol‐aptamer sensor surface.

From the results, it can be inferred that the WATCH sensing platform is sensitive to the glucose and cortisol levels in sweat. The results from the BA analysis highlight that the sensor performance is comparable to the commercial standard techniques. The Pearson's correlation values also highlight the agreement between the standard reference and WATCH results (Figure S2a,b, details presented in the Supporting Information section). The WATCH performance observational study clearly predicts a low‐high‐low biomarker level fluctuation that occurs throughout the day over the period of 8 h 36 min, which is the expected trend in BG levels of healthy individuals per day. The results for sweat cortisol also showcase a similar trend of low‐high‐low (ng/ml). We observed a good correlation in the output data from reference versus sweat sensing studies. Our work presents a three‐way correlation based on WATCH device continuous data output on 10 * 4 individual samples (n = 10 at 4 timepoints), thereby, achieving predictive prognosis in an asymptomatic environment where early disease diagnosis makes a difference, especially in the case of under‐expressed concentrations. A sedentary lifestyle alters the normal secretion of markers that are found in human biofluids. These distortions in normal secretion over time is due to internal immune resistivity, nutrient insufficiencies, hormonal imbalances, less physical activity, and so on, which in turn conceals the signaling efficiency of the metabolic markers. To address the challenge of under‐expressed concentrations, we chose low concentration ranges for sweat cortisol (1–12 ng/ml) and sweat glucose (1–11 mg/dl) markers for performing these studies. Our WATCH data‐driven attempt was to understand sweat glucose (glycemic index profile) and sweat cortisol (adrenal stress index profile) level's relation to human metabolism and food intake by using a single platform for dual‐marker sensing. The grounds for our sweat data sampling and experimentation are a collective effort in emphasizing the platform's ability to map cortisol and glucose simultaneously in sweat samples at discrete time points and correlate it with dietary intake. This data were recorded over 8 h in 10 human participants (n = 10) using the WATCH device. This is a first demonstration to understand the relationship between dietary intake (% of carbohydrates, fat, protein, etc.) and glucose release levels (glycemic Index) and cortisol release levels (adrenal index) simultaneously and continuously in human sweat. The MARD analysis confirms the association of macronutrient consumption to the changes in glucose levels. For the sweat trends highlighted in Figure 5a,b, we observed an approximate overlapping phenomenon in the curves depicting Reference measurements and WATCH measurements for all the discrete time points. This indicates a good agreement between the output results across the three modes of measurement. These results truly highlight the ability of the WATCH sensor to track dual biomarkers sensitively, regardless of the experimental environment it is tested in. This comparison highlights the accuracy and sensitivity of the WATCH sensor performance. It also offers precise insight into the ability of WATCH sensing platform to report these biomarker levels associated with user profiles. Also, it helps with understanding the direction of future case studies that will be performed on significantly larger cohort of samples.

Our research group in collaboration with EnLiSense has demonstrated a continuous and wearable sweat marker sensing technology for tracking dual biomarkers with low detectable concentrations. There are numerous portable sensing devices that are available in the medical device industry as of now with several drawbacks, which are inevitable and need to be addressed. This technology has a great impact on the field of disease diagnosis based medical devices with regards to their performance, validation, and development for clinical application.

## 4 MATERIALS AND METHODS

### 4.1 Reagents and instrumentation

Phosphate buffered saline (PBS) at pH 7.3 and the cross‐linker DTSSP (3,3′‐dithiobis [sulfo‐succinimidyl propionate]) were purchased from Thermo Fisher Scientific (Waltham, MA). The high‐performance liquid chromatography (HPLC) purified 85‐mer cortisol binding ssDNA aptamer (100 ng), Hydrocortisone (corticosteroid) stress hormone, and IDTE resuspension buffer (10 mM Tris and 0.1 mM EDTA at pH 8.0) were purchased from Integrated DNA Technologies (Coralville, IA). The glucose oxidase enzyme (synthesized from *Aspergillus niger*) and D‐(+)‐glucose analyte was purchased from Sigma‐Aldrich Chemicals (St. Louis, MO). In‐house‐made synthetic sweat at pH 7.2 was used for sensor characterization.^39^ The Roche Accu‐check Gluco‐meter was purchased from Amazon.com, Inc. (Seattle, WA) for BG testing.^40^, ^41^ Standard chemical analyses like the Enzyme‐linked immunosorbent assay method (ELISA) and LUMINEX were applied as reference methods. The ELISA kit was a customized kit purchased^42^ from Fab‐Gennix International Inc. (Frisco, TX) and LUMINEX‐xmap technology‐based Cortisol Human Procarta Plex ^(^™^)^ Simplex Kit was purchased^43^ from Thermo‐Fisher Scientific, (Waltham, MA). These reference tests were chosen to compare the WATCH sweat sensing device performance to commercially available technology.

### 4.2 Sweat sensor experimental details

The EnLiSense WATCH device comprises a wearable electronic reader and a replaceable sweat sensing strip that is prefunctionalized for the specific detection of target biomarkers. This is mounted onto the reader that transduces the impedance values from the sensor and outputs a calibrated concentration of the measured biomarker levels in sweat. The sensor fabrication process has been described in detail in our previous work.^44^, ^45^ Nonfaradaic electrochemical impedance spectroscopy (EIS) was used to measure the sensor response to the presence of the target analytes in sweat sampled every minute.^46^ Additional fabrication details and sensor functionalization protocols have been provided in the supplementary section.

### 4.3 Human subject sampling, handling, and storage

Human sweat and blood samples were collected at four discrete time points to gauge not only the glycemic index fluctuations related to their food consumption, but also to predict the cortisol release related stress pattern in a diurnal cycle. Sweat samples were collected from 10 adult healthy human subjects. The collection, processing, and storage was in‐compliance with the protocol approved by the institutional review board at the University of Texas at Dallas (IRB #19–146). Written informed consent was obtained from all the volunteers before experimentation. All 10 healthy cohort samples were collected at time points T1 (before breakfast) around 8:30 a.m., T2 (after breakfast) around 9:30 a.m., T3 (after lunch) around 1:30 p.m., and T4 (late evening) around 5:30 p.m. The collected sweat samples were stored at −20°C for further analysis. Each sweat sample that was collected at different time points was aliquoted. The demographics of the 10 human participants is summarized in Table S2. Our entire experimental results and discussion sections are based on data correlation between standard reference output to WATCH device output. The results from the standard reference method were used to perform correlations for the performance of our sweat marker sensing technology. For sweat glucose measurements ELISA is the reference method. These WATCH measurements were taken using a human subject by placing a sweat sensor on their arm, which includes physical movement. The same experimental process was followed for sweat cortisol measurements with LUMINEX as the reference method and WATCH device as sensing technology. The two modes of measurements were performed on the same sample which was collected from each human subject (˷75–80 μl sample volumes).

## 5 CONCLUSION AND FUTURE WORK

The technology presented in this work, WATCH, is an attempt at understanding the physiological responses to dietary triggers by using a sweat‐based wearable platform that tracks the endocrinal and metabolic indices, cortisol, and glucose. The ability to track glucose accurately (>95–98% accuracy in assay) through sweat without compromising on the sensor's sensitivity and selectivity is challenging and cumbersome. Commercially available BG monitors^47^ are designed to operate within wider concentration profiles, making the detection sensitive, and easy to calibrate. However, in the case of sweat based biomarkers, these concentrations are significantly lower in magnitude. The scale for BG range is 125–285 mg/dl or 7.0–16.0 mmol/L, whereas for sweat glucose (SG), this range goes down to 1.2–25 mg/dl (0.065–1.6 mmol/L); almost 10‐times the magnitude difference in concentrations. Similarly, the blood cortisol levels in healthy humans are anywhere between 5 and 25 μg/dl or 140 and 690 nmol/L,^48^ whereas sweat cortisol levels lie between 10 and 180 ng/ml or 25 and 450 nmol/L.^49^ Tracking these low (low mg/dl or ng/ml) ranges of biomarkers continuously with high precision and accuracy requires sensor devices that perform sensitively but do not compromise on other parameters such as robustness, ability to work with low volumes, quick run time, portability, and noninvasiveness. However, this remains a challenge that needs innovative technological interventions. Toward addressing this challenge of detecting lower ranges of biomarker concentrations (detection limit >2 mg/dl and >2 ng/ml) in a constrained sample volume scenario, we have developed and demonstrated a highly sensitive wearable sweat‐sensor device that can detect using ultralow volumes (1–2 μl) of sweat.

In this study, we demonstrated detection of metabolic markers, sweat glucose in the range of 1–10 mg/dl and sweat cortisol in the range of 1–11 ng/ml in healthy human subjects, both reported simultaneously and continuously using the WATCH device. The sweating abnormalities due to a lack of thermo regularity and metabolic balance makes it critical to monitor these biomarkers, especially in prediabetic conditions to track the slightest elevation in glucose levels^50^ for a better prognosis. We provide the results of a cross‐sectional observational study where we demonstrate the feasibility of evaluating temporal trends of these metabolic biomarkers over an 8‐hr period using wearable technology that works with passively expressed sweat. As a pilot study, we employed healthy humans as test subjects, in a way that allows for detection of lowest possible concentrations and confirm the efficacy in terms of sensitivity and specificity of our sensing device. We also demonstrate the feasibility to correlate and assess macronutrient subgroups and determine their relationship with glucose and cortisol in sweat. Hence, this work paves the path for the future outlook of providing a better and comprehensive disease related sample analysis. We plan to further expand this work in future prospective studies where there is controlled nutrition consumption to better understand and decouple the relationship between a specific macronutrient group consumption and its impact on glucose and cortisol release rates in varying real‐world environments.

## CONFLICT OF INTEREST

Drs. Shalini Prasad and Sriram Muthukumar have a significant interest in EnLiSense LLC, a company that may have a commercial interest in the results of this research and technology. The potential individual conflict of interest has been reviewed and managed by the University of Texas at Dallas and played no role in the study design; in the collection, analysis, and interpretation of data; in the writing of the report, or in the decision to submit the report for publication.

## AUTHOR CONTRIBUTIONS

**Madhavi Pali, Badrinath Jagannath, Kai‐Chun Lin**: Methodology, resources, investigation, validation, data curation, writing—original draft, and visualization. **Devangsingh Sankhala**: Resources, methodology, validation, data curation, investigation, writing, and visualization. **Sayali Upasham**: Writing—review and editing. **Sriram Muthukumar and Shalini Prasad**: Conceptualization, resources, writing, and review & editing.

### PEER REVIEW

The peer review history for this article is available at https://publons.com/publon/10.1002/btm2.10241.

## Supporting information

**Appendix** S1: Supporting Information

## Data Availability

The data that support the findings of this study are available from the corresponding author upon reasonable request.
