# Supplementary material for: Tracking metabolic responses based on macronutrient consumption: A comprehensive study to continuously monitor and quantify dual markers (cortisol and glucose) in human sweat using WATCH sensor
Source: Bioeng Transl Med. 2021 Jul 29;6(3):e10241. doi: 10.1002/btm2.10241 (PMC8459601; doi:10.1002/btm2.10241)
Supplement: Supplementary file 1 — Appendix S1: Supporting Information [file BTM2-6-e10241-s001.docx]

**Tracking metabolic responses based on macronutrient consumption:**

**A comprehensive study to continuously monitor and quantify dual markers (Cortisol and Glucose) in human sweat using WATCH sensor**

Madhavi Pali^a^, Badrinath Jagannath^a^, Kai-Chun Lin^a^, Devangsingh Sankhala^b^, Sayali Upasham^a^,

Sriram Muthukumar^c*^, and Shalini Prasad^a*^

^a^ Department of Bioengineering, University of Texas at Dallas, Richardson, TX-75080

^b^ Department of Electrical Engineering, The University of Texas at Dallas, Richardson, TX, USA 75080

^c^ EnLiSense LLC, 1813 Audubon Pond Way, Allen, TX, USA 75013

* Corresponding Author email: [shalini.prasad@utdallas.edu](mailto:shalini.prasad@utdallas.edu) ; [sriramm@enlisense.com](mailto:sriramm@enlisense.com)


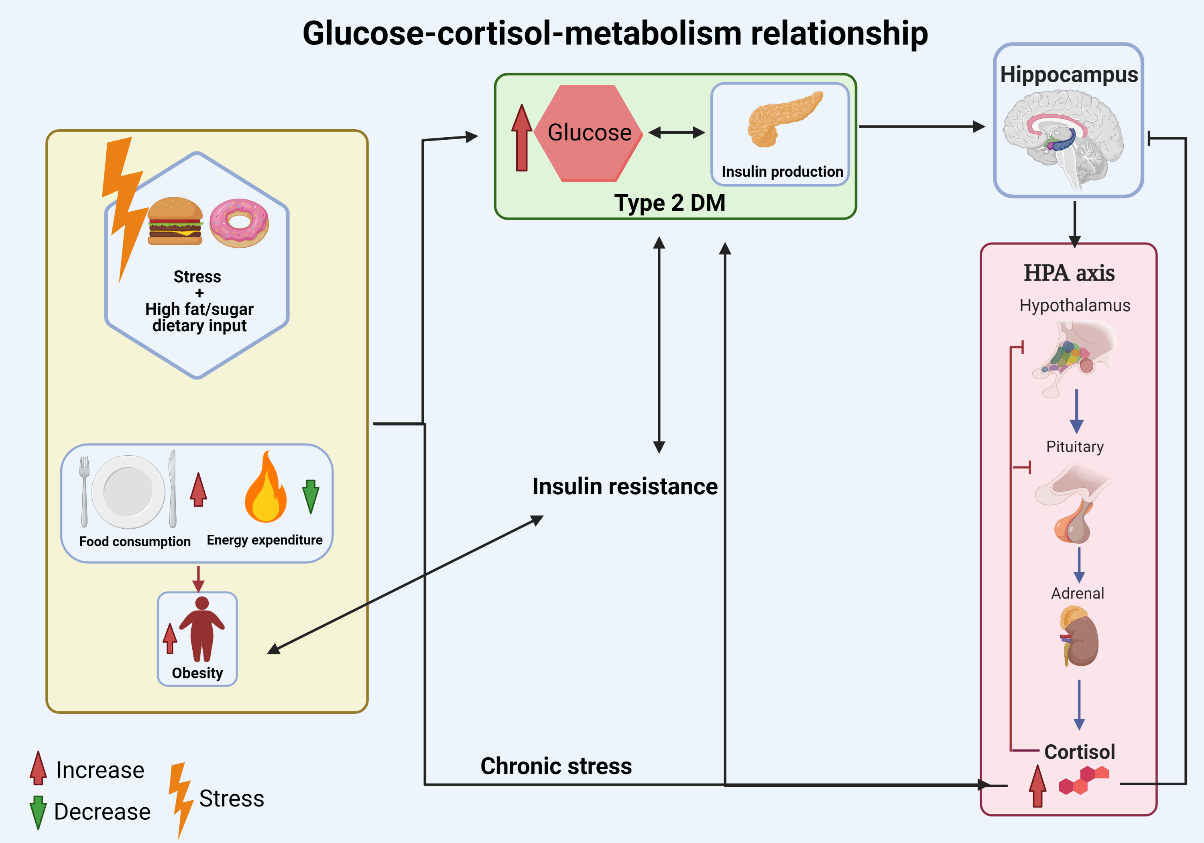


Figure S1: Relationship between glucose-cortisol-metabolism highlighting the interconnectivity for chronic stress and diet induced diseases

**Table S1**: Summary of the sweat glucose and cortisol levels for different time points

| Concentration ranges | T1-Timepoint | | T2-Timepoint | | T3-Timepoint | | T4-Timepoint | |
| --- | --- | --- | --- | --- | --- | --- | --- | --- |
|  | ELISA | WATCH | ELISA | WATCH | ELISA | WATCH | ELISA | WATCH |
| Sweat Glucose levels (mg/dL) | 0.09-3.31 | 0.60-4.20 | 1.01-9.10 | 0.85-9.35 | 0.95-4.20 | 1.05-5.30 | 0.80-5.85 | 1.01-5.75 |
|  | LUMINEX | WATCH | LUMINEX | WATCH | LUMINEX | WATCH | LUMINEX | WATCH |
| Sweat Cortisol  levels (ng/mL) | 1.88-5.63 | 1.65-12.45 | 1.58-8.27 | 3.23-9.25 | 2.09-5.76 | 3.15-9.15 | 1.40-5.23 | 2.11-10.35 |

**Table S2:**Characteristic table of 10 human subject cohort sweat sample information with obtained average concentrations for sweat Glucose-Cortisol study

|  | Number of participants (n=10) |
| --- | --- |
| Age range (years) | 24-40 |
| weight range (kgs.) | 59-95 |
| Height (meters) | 1.44-2.79 |
| BMI | 25-32 |
| Race | Asian (6), Caucasian (3), South American (1) |
| gender | Male (6), Female (4) |
| Blood glucose average (mg/dL) | 105.5±8.3 |
| Sweat glucose average (mg/dL) | 5.5±3.2 |
| Sweat cortisol average (ng/mL) | 11.1±2.5 |
| Sampling mode | Blood-Finger prick |
|  | Sweat-IRB-UTD |
| Collected sweat volume range (µL) | 68-130 |
| *#* on antibiotics medication | none |

The table includes their measured blood glucose, sweat glucose, and sweat cortisol levels. Since all the participants are healthy individuals, we can see the range which they fall under is quite close to normally presented glucose and cortisol levels in healthy human sweat. These marker concentration ranges are in-agreement with literature available data (Hogenelst, Soeter and Kallen, 2019) (Jones *et al.*, 1995).

**Sweat sensor device and Peripherals**

The reader also comprises of a temperature and RH sensor (Texas Instruments Inc., USA), a battery management system, and Bluetooth Low Energy (BLE) module integrated with a central ARM core processor. The temperature and RH sensor help measure the body temperature and perspiration at the epidermal surface where the sensor is applied. The Bluetooth Low energy module on the reader allows for wireless communication to a smart device with a customized app to transduce the data from the sensors sampled every minute. The reader is powered with a 3.7 V LiPo battery that has a lifetime of 168 hours on a single charge. The WATCH device fabrication and surface chemistry functionalization was described in detail in previous work from our group (Ganguly *et al.*, 2020).

**Sensor surface-assay functionalization**

The sensor surface for sweat glucose detection was developed by immobilizing the glucose oxidase (G-Ox) enzyme (100µg/mL), which was derived from *Aspergillus niger* fungi (10mg/dL) in a PBS medium (pH 7.2). For the sweat cortisol analyte detection, the sensor surface was functionalized with a 5-primer -SH linked cortisol aptamer-solution-100 nM (1µM DNA oligomer) in IDTE at pH 8.0 medium (1x TE Base). Both the sensor surfaces were functionalized with their respective substrate chemistry (Hermanson, 2013) overnight (˷8 hours) to get a complete covalent bond saturation on the sensor surfaces and stored at 4^°^C. Signal fluctuations due to buffer variations are minimized by performing baseline correction with synthetic sweat at pH 7.2 on assay immobilized sensor surfaces. supplementary All the electrochemical impedance measurements were performed in a non-faradic setting. A low sinusoidal input voltage was applied and the resulted impedance due to the covalent interaction between target analyte and capture monoclonal antibody probe was recorded.

**WATCH Performance: Correlation study**

The Spearman rank correlation is used for understanding the strength and direction of the association between two ranked variables and their monotonic relationship (linear and nonlinear), whereas the Pearson’s ‘r’ describes the linear correlation between two sets of data along with a linear regression r^2^ value. The data presented was distributed in an elliptical manner and shows minimum (less prominent) outliers, leading to similar results in both the correlation studies. The advantage of measuring the association between two ranked/ordered variables, irrespective of their linearity, is that it gives more room to fit data in an asymmetric environment where the method of experimentation is considerably varied by different parameters. For example, like primary and secondary antibody conjugations, signal detectors, testing environment, sample preparation, buffer, and pH optimization, etc. Spearman rank correlation values of the WATCH sensor performance further confirms the reliability of the data and that it aligns with the performance of a standard reference method. This agreement provides us a promising path of applying sweat sensor technology as a carryout, user-friendly, rapid result-based diagnosis supportive device in replacement of a high-end, bulky, time-restricted, operator’s skill-based equipment usage. The following Figure S2a provides the scatter matrix plots of sweat glucose levels in two different measuring environments along with their Pearson’s r value. The sweat glucose Pearson’s r value is 0.94719 for WATCH with respect to the ELISA reference method, and the corresponding r^2^ value is 0.89446. The following Figure S2b provides the scatter matrix plots of sweat cortisol levels in 2 different measuring environments along with their Pearson’s r value. The sweat cortisol Pearson’s r value is 0.86824 for WATCH with respect to LUMINEX reference method, and corresponding r^2^ value is 0.97940. The obtained Spearman rank correlation value is 0.89532 for WATCH results and are a close match to Pearson’s r values in the sweat glucose study. The Spearman rank values of sweat cortisol measurements is 0.83775 for WATCH and these results are in-agreement with this study Pearson’s r values as well. The results for all ranked correlations of sweat glucose and sweat cortisol levels between the two different testing environments is provided in Table S3.

**Table S3**:The Spearmen rank correlation tabulated data of WATCH output with respect to the standard reference method

| Spearman rank correlation | Sweat Cortisol study | | | |
| --- | --- | --- | --- | --- |
|  | **ELISA** | **WATCH** | **LUMINEX** | **WATCH** |
| Reference | **1** | **0.89532** | **1** | **0.83775** |
| WATCH | **0.89532** | **1** | **0.83775** | **1** |





Figure S2: a. Linearly correlated scatter matrix plots for sweat glucose study, a comparison between different experimental environments (ELISA vs. WATCH), b. Linearly correlated scatter matrix plots for sweat cortisol study, a comparison between different experimental environments (LUMINEX vs. WATCH)

**Probability distribution and Power analysis**

For sweat glucose measurements, the probability > |t| value is 5.69051*10^-10^ with an SEM (Standard Error of Mean) difference of 0.255mg/dL for ELISA, and the probability > |t| value is 4.76190*10^-10^ with an SEM difference of 0.26114 mg/dL for WATCH. For sweat cortisol measurements, the probability >|t| value is 3.18011*10^-10^ with an SEM difference of 0.21582 ng/mL for LUMINEX, and the probability >|t| values is 3.17466*10^-10^ with SEM difference of 0.33944 ng/mL for WATCH.
